# Sexual Dysfunctions and Problematic Sexuality in Personality Disorders and Pathological Personality Traits: A Systematic Review

**DOI:** 10.1007/s11920-023-01409-9

**Published:** 2023-02-04

**Authors:** Giacomo Ciocca, Ramona Di Stefano, Alberto Collazzoni, Tommaso B. Jannini, Giorgio Di Lorenzo, Emmanuele A. Jannini, Alessandro Rossi, Rodolfo Rossi

**Affiliations:** 1 Department of Dynamic and Clinical Psychology, and Health Studies, Sapienza University of Rome, Rome, Italy; 2 Department of Biotechnological and Applied Clinical Sciences, University of L’Aquila, L’Aquila, Italy; 3 Renewed Freedom Center for Rapid Anxiety Relief, Division of Strategic Cognitive Behavioral Institute, Los Angeles, CA USA; 4 School of Psychiatry, Department of Systems Medicine, University of Rome Tor Vergata, Rome, Italy; 5 Department of Systems Medicine, University of Rome Tor Vergata, Rome, Italy

**Keywords:** Personality disorders, Personality traits, Sexual dysfunction, Problematic sexuality, Sexual risk behavior, Adverse childhood experiences

## Abstract

***Purpose of Review*:**

This aim of the present systematic literature review is to critically analyze problematic sexuality and sexual dysfunctions in personality disorders (PDs) and pathological personality traits.

***Recent Findings*:**

An initial pool of 123 studies was found, out of which 17 met the selection criteria and were therefore included. Traumatic experiences as childhood sexual abuse and adverse childhood experiences characterize the relationship between sexual behavior and PDs. From this point of view, sexual compulsivity and sexual risk behaviors, typical of BPD and ASPD, respectively, are among the pathognomonic aspects of PDs and of pathological personality traits.

***Summary*:**

A maladaptive personality functioning may manifest through a problematic sexuality and a sexual impairment. In this regard, traumatic life experiences may structure personality together with sexual functioning. Therefore, it would be useful to consider the relationship between trauma, sexuality, and personality in research and in the clinical setting.

**Supplementary Information:**

The online version contains supplementary material available at 10.1007/s11920-023-01409-9.

## Introduction

Personality disorders (PDs) can be defined as a pattern of behavior that negatively affects the life of an individual [1]. This pattern is pervasive, stable, firstly appearing during adolescence and the first adult age. People with PDs suffer intensely and dramatically, finding difficulties in conducting and developing a normal life and in creating satisfactory and adequate relationships.

In this regard, PDs can be defined as disorders of human significant relationships, in which lies the psychological suffering. This aspect has been highlighted in the Diagnostic and Statistical Manual of Mental Disorders (DSM-5) [2].

Personality functioning is determined by several variables and critical life events. Among these, primary relationships and traumatic experiences play a pivotal role for the development of personality [3, 4]. In this regard, an interesting perspective, explaining the development of pathological personality, was theorized by Kernberg and his theory of objectual relationships [5]. This contemporary psychodynamic perspective includes the central role of intimacy in the personality functioning, highlighting the concept of capacity to love and its related limitations [6]. This central aspect of psychic functioning may be considered as an indicator of mental health to discriminate normal from pathological personalities [7].

In personalities where paranoid, narcissistic, and masochistic traits are marked, limitations to the capacity to love may occur [6]. The capacity to love, related to the intimacy dimension and to the ability to engage a profound relationship, recalls another central aspect of the psychic functioning, i.e., the sexual behavior.

Human sexual behavior is the result of biological, socio-cultural, psychological, relational, and evolutionary factors, and its clinical perspective is mainly focused on the neuroendocrinological and psychological aspects [8]. In this regard, personality functioning plays a central role in sexual behavior and in cases of problematic sexuality or sexual dysfunctions [9].

Many facets of problematic sexual behavior have been found to be associated with pathological personality or dysfunctional personality traits, and, in some cases, sexual problems can be considered symptomatologic manifestation of PDs, per se. Likewise, specific etiological factors determine personality and sexological dysregulation at the same time [10, 11, 12].

For instance, sexual risk behaviors (SRB) are often associated with borderline personality disorders (BPD), typically but not exclusively in youth [13], while a propensity to marital infidelity may characterize narcissistic subjects [14]. Moreover, a particular form of dependence, like sexual compulsion, is frequently comorbid with paranoid, narcissistic, antisocial, avoidant, obsessive–compulsive, and passive-aggressive personalities, although with different prevalence [15]. Both paranoid and obsessive–compulsive PDs share a high rate of divorces, demonstrating the central role of suffering personality in the quality of couple’s life [16].

A large part of literature on sexuality and personality studied the borderline area. BPD is the best representation of a suffering personality where traumatic life experiences, together with an insecure attachment style, compromise adult relationships as well as sexual behavior. In this regard, sexual behavior and sexual functioning are central indicators of psychological wellness and of general mental health. Therefore, in light of the strong association between personality and sexuality, the contemporary psychopathology considers sexual functioning as the result of personality functioning.

The aim of the present article is to analyze, through a systematic review of the literature, the association between problematic sexuality, sexual functioning, and personality traits and disorders.

## Methods

This systematic review of the literature was conducted following the Preferred Reporting Items for Systematic Reviews and Meta-Analyses (PRISMA) guidelines [17]. Articles were retrieved via the electronic databases “MEDLINE” (National Library of Medicine–Bethesda USA) and PsycInfo, by two all-term searches for each of the two selected databases. Each of the two selected databases was searched via the terms “personality disorder” and “sexual dysfunction” joined by the Boolean operator AND. A second search strategy was performed on the same databases via the terms “personality disorder” and “sexual risk behavior,” joined by the Boolean operator AND.

Search period was from 1 January 2019 to 25 April 2022. The complete search line as processed by the search engines is available in the supplementary material in Online Resource 1.

### Inclusion Criteria

To be included, articles had to be identifiable by searching the abovementioned databases and be fully published on scientific journals after being peer-reviewed. Search was restricted to the English language. Studies concerning sexual dysfunction and SRB as related to PDs and personality traits were considered relevant to our search and therefore included, unless having at least one exclusion criterion.

### Exclusion Criteria

We excluded those studies which were defined as not relevant to the study topic. Furthermore, all non-peer-reviewed studies were excluded. Design studies such as “abstract,” “letter,” “commentary,” “short communication,” “feature article,” or “personal view” were excluded as well.

### Article Selection and Data Extraction

Two authors (RDS and GC) independently performed the search on each of the selected computerized database, generating a list of retrieved articles. The said list was then entered on a computerized master log, on which the very first selection of studies was performed according to the topic relevance by reading the title and abstract only. Without consulting, each of the two authors performed a second screening of the so-compiled article list, in order to identify relevant studies by reading each article full text. Only at this time did the authors consulted each other to resolve conflicting decisions about included and excluded articles. When conflicts were reported, a third author (RR) was consulted. After a final included list was obtained, data extraction took place by entering abstracted information in a computerized database. Abstracted information included publication title, first author, journal name, year of publication, study design, primary objective of the study (if applicable), characteristics of included population (if applicable), scales and measures utilized to evaluate the study outcome (if applicable), and main results. AC, TBJ, GDL, EAJ, and AR contributed to the “Results” and “Discussion” sections.

### Quality Assessment

The quality of observational studies was evaluated independently by RDS and GC using Newcastle Ottawa Scale (NOS). Discrepancies in selection were solved by confrontation. Studies found to be non-satisfactory according to NOS tool were excluded.

## Results

### Study Selection

**Fig. 1:**
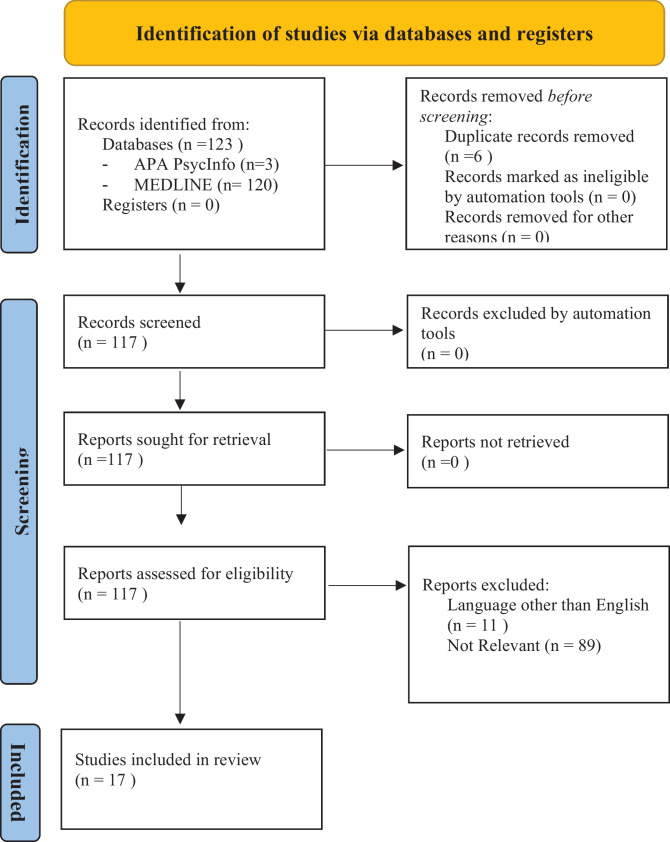
PRISMA 2020 flow diagram of included studies

The database search identified 123 records. After the removal of 6 duplicates, 117 studies were screened for title and abstract. The screening process led to 101 studies excluded. The full text of the remaining studies was assessed. Finally, 17 studies were included into the database of the present review following the aforementioned inclusion criteria. The study selection process is depicted in Fig. 1 (PRISMA).

All the included studies were observational studies published in a period from 1 January 2019 to 25 April 2022. The main characteristics of the analyzed studies are summarized in Table 1.

**Table 1:** Main findings and characteristics of included studies

First author	Journal	Study design	Tested outcomes	Number and characteristics of sample	Scales and measures	Main findings
Hemmati et al. 2020 [18]	Indian J Psychol Med	CS	Determine the reflection of childhood sexual abuse (CSA) on the pathological traits of the alternative model of personality disorders (AMPD) in section III of DSM-5 and Cloninger’s temperament and character profiles	433 Iranian college students. 390 without a CSA experience (mean age 27.15 years, 31% female) and 43 with a CSA experience (mean age 24.30 years, 53.5% female)	Identification of CSA; Personality Inventory for DSM-5 (PID-5)-Persian Version; Temperament and Character Inventory (TCI-125)-Persian Version; Structured Clinical Interview for DSMIV-TR-Axis II-Screening Questionnaire (SCID-II-SQ)36-Persian Version	Both PID-5 and TCI-125 were significantly associated with their relevant PDs. Pathological trait domains of the PID-5, negative affectivity excluded, were greater in those with a CSA experience. Novelty seeking and persistence dimensions of the TCI-125 were higher in CSA experience population
Kahn et al. 2020 [19]	Sexual Abuse	LS	Understanding of the frequency of adverse childhood experiences (ACE) in a sample of high-risk civilly committed sexual offenders and the link between ACE scores and diagnoses of adult pshycopathology	317 adult males who had committed sexual offenses. Mean age 51.38 years; 69.1% Caucasian	ACE scale; Static-99R; DSM-5 Diagnoses	ACE score-sexual abuse item was present in 56.8% of the examined population. Total ACE scores were significantly related to a diagnosis of antisocial personality disorder (ASPD), with the odds of having ASPD increasing by 11% for each additional ACE item endorsed
Ajo et al. 2020 [20]	Int J Impot Res	LS	Analysis of the influence of PD in patients with erectile dysfunction (ED) and chronic non-cancer pain (CNCP) and their response to andrological treatment	62 male patients with ED and CNCP (mean age 57 years)	Millon Clinical Multiaxial Inventory (MCMI-III); International Index of Erectile Function (IIEF)	31% of the sample presented a base rate ≥ 85 scores at the MCMI-III Questionnarire (determine the presence of the PD or clinical syndrome). The presence of features of several PDs and clinical syndromes was significantly associated with a worst sexual life quality, anxiety, and depression. Self-defeating feature presence was significantly correlated with a more severe baseline ED and narcissistic with a better response to andrological treatment
Ballester-Arnal 2020 [21••]	Addictive Behaviors	CS	The study is aimed at exploring psychiatric comorbidity in a sample of individuals with and without compulsive sexual behavior disorder (CSBD)	315 participants low-CSBD risk profile (mean age 20.89 and 54.9% females); 68 participants with high-CSBD risk profile (mean age 20.63 and 33.8% females)	Hypersexual Behavior Inventory (HBI); Sexual Compulsivity Scale (SCS); Sexual Addiction Screening Test (SAST); SCID-I; SCID-II (only Borderline BPD-BPD and Obsessive–Compulsive PD-OCPD were explored in this study)	5.9% of the CSBD subjects presented a borderline personality disorder (BPD) compared to 0.3% in non-CSBD subjects
Miano et al. 2020 [22]	J Pers Disord	CS	Characterization of interpersonal relationships of patients with BPD	31 heterosexual couples with BPD women (mean age 31.84 years) and 36 healthy control couples (mean age 30 years)	SCID-II; Mini International Neuropsychiatric Interview (MINI); relationship stability questions from Kenny and Acitelli; Partnership Questionnaire (PFB-K); the Problem List (PL) of relationship conflict; Dependency and Insecurity Scale (DSC); Relationship Questionnaire (RQ-2)	42% of BPD females signaled “attention of partner” and “sexuality” as unresolved conflict areas, compared to HC couples that reached 14% and 17% respectively in these areas
Beauregard and DeLisi 2021 [23•]	J Interpers Violence	CS	Examination of the personality profile of the Sexual Homicide Offenders (SHOs)	85 SHO; 144 Violent Non-Homicidal Sex Offenders (VNHSOs); 387 Non-Homicidal Sex Offenders (NHSOs)	PDs assessment using DSM-4; Minnesota Multiphasic Personality Inventory-II (MMP-II); Psychopathy Checklist-Revised (PCL-R)	The study reported a personality profile correlation of SHOs with Schizoid and BPD. SHOs were likely to select a victim, use a weapon, and use drugs and alcohol before their offenses
Bégin et al. 2022 [24••]	Frontiers in Psychology	LS	Identification of distinct risky sexual behaviors (RSB) profiles in youth and their association with BPD features	126 adolescents and young adults (41 aged 14–17 and 85 aged 18–21; 82% females)	Sexual Risk Survey (SRS); Borderline Personality Features Scale for Children (BPFS-C)	3 distinct RSB profiles were identified: (1) low RSB profile; (2) Unprotected Sex in Relationships profile; (3) impulsive sex outside relationships profile. The third profile was manifested by youth with significantly higher BPD features
Natoli et al. 2021 [25]	Archives of Sexual Behavior	LS	Link between interpersonal dependency and sexual and romantic relationships and sexual activity	30 self-identified females and 30 self-identified males	Rorschach Oral Dependency Scale (ROD); self-report measure of interpersonal and healthy dependency; Structured Interview of Personality Organization (STIPO); Relationship Profile Test (RPT)	A dependent personality style was found to be positively associated with one’s likelihood of being in a romantic relationship, but was not found to alter the likelihood of being sexually active
Wetzel et al. 2019 [26]	Self and Identity	LS	Investigation of developmental links between narcissism, problem behaviors, and adolescence	674 Mexican-origin adolescents at 14 and 16 years of age	Narcissistic Personality Questionnaire for Children- Revised (NPQC-R); Assessment of delinquency with a scale from the National Longitudinal Study of Adolescent Health (ADD Health); Drug use assessment with a count score of 7 items adapted from the National Youth Survey; two questions assessing sexual behavior: “Have you had sexual intercourse during the past 12 months?” and “How many people have you had sexual intercourse with during the past 12 months?”; Diagnostic Interview Schedule for Children (DISC-IV)	Results showed that adolescent exploitativeness at age 14 prospectively predicted sexual behavior at age 16, which provides initial evidence for an association between narcissistic tendencies and sexual behavior in adolescence, suggesting that both behavioral and affective features of narcissistic exploitativeness may influence the occurrence of early sexual behavior among youth
Choukas-Bradley et al. 2020 [27]	J Abnorm Child Psychol	LS	Examination of developmental trajectories of adolescent girls’ BPD symptoms and sexual risk behavior (SRB)	1620 Black and White girls from the Pittsburgh Girls Study	BPD screening questionnaire of the International Personality Disorders Examination (IPDE-BOR); Adolescent Sexual Activity Index (ASAI); Adolescent Symptoms Inventory-4 (ASI-4)	Girls with more BPD symptoms at age 14 showed steeper growth over time in SRB from ages 14 to 18. Additionally, adolescents who showed steeper increases in BPD symptoms over time also showed steeper increases in SRB across adolescence
Penner et al. 2019 [28]	Personal Dis	LS	Evaluation of RSB and correlates of RSB among female adolescent inpatients with and without BPD	123 females (mean age 15.5 years) inpatient in a psychiatric unit for adolescents. 50 participants met full criteria for DSM–5 Section II BPD and thus comprised the BPD group (mean age 15.04 years)	Safer Choices Survey (SCS); Sexual Risk Behaviors Beliefs and Self-Efficacy Scales (SRBBS); Childhood Interview for BPD (CI-BPD); Borderline Personality Features Scale for Children (BPFSC); Youth Self-Report (YSR)	The BPD group evidenced significantly lower self-efficacy to refuse sex, and riskier attitudes. This may influence adolescent girls with BPD to engage to higher levels of RSB later in life
DeLisi et al. 2019 [29••]	Compr Psychiatry	LS	Evaluation of the etiology of ASPD in terms of ACE and childhood psychopathology	863 active correctional clients (mean age 44 years)	DSM-4 diagnosis; ACE events	Greater ACEs were associated with ASPD diagnosis with physical abuse showing association with ASPD symptoms and sexual abuse with lifetime diagnosis for ASPD

Key: *LS*, longitudinal study; *CS*, cross-sectional study.

### Adverse Childhood Sexual Experiences as Predictive Factor of Personality Disorders

A study by DeLisi and colleagues points out that having a sexual trauma during childhood predicts the development of a future PD, besides the well-known link with psychotic spectrum disorders [29••]. Accordingly, Hemmati and colleagues investigated the relationship occurring between childhood sexual abuse (CSA) and pathological traits of the alternative model of personality disorders (AMPD) in section III of DSM-5, evaluating a sample of 433 Iranian college students. Forty-three individuals in the sample experienced a CSA. The study showed greater pathological trait domains of the Personality Inventory for DSM-5 (PID-5) in individuals with a CSA experience [18]. Similarly, Flynn and colleagues analyzed the association between adverse childhood experiences (ACEs), PDs symptoms and health risk behaviors (HRB). ACEs positively predicted the latent HRB variable and PD symptomatology. At high levels of ACEs, childhood resiliency did not function as a protective factor for HRBs and PD symptoms [30].

In a study by Kahn et al., 317 adult males who had committed sexual offenses were analyzed to verify the frequency of ACE in their history. A statistically significant correlation was found between total ACE scores and the diagnosis of antisocial personality disorder (ASPD). The odds of having ASPD increased by 11% for each ACE item additionally reported [19]. Likewise being physically abused during childhood predicted the onset of an ASPD [29].

### The Effect of Personality Disorders on Sexual Functioning

Investigating the key role of personality in sexual functioning, Ajo et al. pointed out how dysfunctional traits may influence patients’ sexual health.

The study observed the influence of PDs on the response to andrological treatment of 62 male patients with erectile dysfunction (ED) and chronic non-cancer pain (CNCP). The patterns of PDs were investigated using the Millon Clinical Multiaxial Inventory (MCMI-III). The presence of features of PDs was significantly associated with worse sexual life quality, anxiety, and depression. Self-defeating feature was correlated with more severe ED, while individuals with narcissistic features showed a better response to andrological treatment [20].

Miano and colleagues conducted a cross-sectional study about the interpersonal relationships in individuals affected by BPD. Scales were administered to 31 heterosexual couples with BPD women and 36 healthy control (HC) couples. Among involved BPD women, 42% indicated “sexuality” as an unresolved conflict area, compared to 17% signaled by HC couples [22].

A study on 509 Polish women showed a correlation of personality types with sexual excitation (SE) and sexual inhibition (SI). More extraverted women reported higher levels of SE, while women with higher level of neuroticism reported greater SI [31].

Sanchez et al. performed an evaluation of the prevalence of high-risk sexual behaviors (HRSB), their consequences, and associated factors in 103 young individuals attending a personality disorders clinic. Among them, a total of 77.7% already was sexually active, 37.5% did not use appropriate contraceptive methods, and 25.5% tested positive for a sexually transmitted infection. Nevertheless, the study found no significant association between HRSB and clinical and demographic characteristics or symptoms severity [32].

An observational study conducted on 656 women is aimed at evaluating the association of the self-reported pathological narcissistic traits with sexual functioning. Results showed lower sexual functioning in individuals with vulnerable narcissistic traits and higher levels of body image self-consciousness. Conversely, grandiose narcissistic traits appeared to be linked to higher levels of sexual functioning and lower image self-consciousness [33].

### Personality Disorder Correlation with Sexual Functioning

A cross-sectional study by Ballester-Arnal et al. explored the psychiatric comorbidities of patients affected by compulsive sexual behavior disorder (CSBD). Study found a correlation between BPD and CSBD risk profile. In the examined cohort, a high CSBD risk profile was correlated with BPD in 5.9% of participants, compared to a 0.3% in the low-risk cohort [21••].

The personality profiles of Sexual Homicide Offenders (SHOs), Violent Non-Homicidal Sex Offenders (VNHSOs), and Non-Homicidal Sex Offenders (NHSOs) were described in a study by Beauregard and DeLisi. In the study, researchers reported a link between schizoid and BPD and being a SHOs. VNHSOs are more likely to present Antisocial and Paranoid PDs instead. Finally, the NHSOs presented more frequently Avoidant and Dependent PD profiles [23•].

The association between distinct risky sexual behavior (RSB) profiles and BPD features in youth was investigated by Bégin et al. in a longitudinal study on 126 adolescents and young adults. This research found significantly higher borderline personality pathology levels in participants with impulsive sex outside relationships profile [24••].

Natoli and colleagues investigated the correlation between dependent personality profile and sexual relationships and activity. The work showed lower self-reported interpersonal dependency scores and higher healthy dependency scores in sexually active individuals and in individuals with past sexual activity. However, dependent personality style seemed to not alter the likelihood of being sexually active and is positively associated with likelihood of being in a romantic relationship [25].

The association between PDs and sexual activity and behavior in adolescents was investigated by various studies. Weltzel et al. observed the developmental link between narcissism, problematic behaviors, and adolescence in a longitudinal study on 674 adolescents aged 14 and 16 years old. Adolescent narcissistic exploitativeness at age 14 prospectively predicted sexual behavior at age 16. Adolescents with higher exploitativeness levels were more likely to have sexual intercourse and exhibit symptoms of conduct disorder than adolescents with lower levels [26]. Two other studies examined the link between BPD symptoms and SRB in a population of adolescent girls at different ages. Girls with higher levels of BPD symptoms at age 14 exhibit a greater and more precipitous overtime growth in SRB. Among them, those with steeper increase in BPD levels also showed more abrupt increase in SRBs across adolescence. Additionally, BPD symptoms seemed to be associated with heightened relational insecurity across adolescence [27, 34•]. Moreover, lower self-efficacy to refuse sex and riskier attitudes were found in the BPD group of participants in a study by Penner and colleagues [28].

## Discussion

The major novelty found in this systematic review is the relationship between sexual symptoms, PDs and pathological personality traits. Similarly, psychotic spectrum disorders have been found to affect sexual functioning [35, 36, 37]. While in some cases, sexual symptoms may predict mental diseases, the same link in PDs is still not fully understood. However, the reduction in sexual health experienced in PDs should be considered in evaluating pre- or sub-clinical cases of psychotic spectrum disorders [38]. The transition toward psychosis, in fact, could be predicted by several dysfunctional personality traits, as schizotipy or paranoid ideation, that could affect sexual behavior [38, 39, 40].

Traumatic life experiences may be considered as a common factor in mental suffering and the psychopathology of sexual behavior. Trauma is, in fact, considered the main predictor of psychosexological suffering, as in sexual compulsivity or hypersexuality [11]. The systematic search of literature reveals the central role of traumatic events in the psychosexological functioning and, as several evidence demonstrated, sexual abuse is a dramatic and central factor modulating PDs [41]. Similarly, ACEs have a pivotal role to determine PDs and deviant behavior in sexual offenders [19]. The theory of abused-abusers in sexual offenders appears confirmed also in PDs, such as the antisocial behaviors [42]. This aspect reinforces the defining characteristic of trauma and traumatic life experience in personality and in its development. Sexual abuse and ACE base the development of personality on traumatic elements. The traumatic personality is able to impair the committed relationships and the intimacy, as well as the sexual behavior leading to SRB and sexual compulsivity [43].

Moreover, literature shows that PD type and severity influence sexual functioning and behavior, according to the type of PD [9]. The PD influences self-perception, body image self-appreciation, partner-related romantic, and sexual relationships, playing an important role on different phases of sexual arousal and inhibition [22, 31, 32, 33]. People with Cluster A PD are indeed not interested in creating relationships, therefore, being incapable of having a healthy sexual life. Cluster B PD are instead characterized by having a more turbulent sexuality in light of their disorganized attachment style. For instance, some PDs appear to be strongly connected to an increased risk of engaging HRSB and a lower self-efficacy in refusing sex, when offered the chance [28, 32]. BPD individuals show higher levels of impulsivity and higher chance of being involved in risky behavior with respect to the healthy population. These peculiar traits are reflected on sexuality of BPD affected individuals who show impulsivity in engaging sexual intercourses and manifest RSB [24••, 27, 32, 34•]. Moreover, sexuality could be interpreted by BPD patients as a constant unresolved conflict area in the couple [22]. Data reviewed here highlighted the influence that PDs and pathological personality traits could have on the treatment for a SD, such as ED [20]. This finding suggests the importance of an appropriate investigation of PD profiles approaching to diagnosis and treatment of SDs, an attitude not always present in mental health professionals [44].

As a part of our review, we tried to elucidate the role of PDs in engaging different sexual behaviors. Recent literature correlates the presence of PDs with several dysfunctional sexual behaviors such as compulsive sexual behavior, RSB, sexual offenses, and impulsive sex outside relationships [21••, 23•, 24••, 27, 28, 34•]. Extensive literature is available on the topic of the correlation of SDs and RSB in BPDs [21••, 24••, 27, 28, 34•]. The emotional and relational instability, the process of idealization and devaluation of self and of interpersonal relationships, and the marked impulsivity correlate with greater difficulties in engaging a healthy and satisfactory sexual relationship, both inside and outside a stable romantic relationship. These patients show more proneness to engage RSB [24••, 27, 32, 34•] and have a greater relational insecurity, also resulting in an earlier sexual debut during adolescence [27, 34•]. Also, individuals with higher levels of narcissistic exploitativeness showed greater risk of an earlier sexual debut [26].

The role of dependent personality disorder (DPD) in the development of sexual relationships was also evaluated. To date, evidence on DPDs shows that these individuals tend not to be markedly impaired in their sexual life, both in terms of likelihood of being sexually active and living a healthy sexual relationship [25]. Nevertheless, although findings on patients with cluster C PDs are lacking, one might hypothesize a dysfunctional sexuality, as the fear to undergo rejections from others may generate feelings of sexual failure and therefore avoidant behaviors over sexual experiences. Indeed, Benotsch and colleagues described high levels of impaired sexual behaviors (having less say in sexual behaviors, having sex because of threats and fear of asking a partner to use a condom) in individuals with marked dependency trait [45].

A correlation with the chance of being affected by different PDs appeared to be present at the base of sexual offense perpetuation, as reported by Beauregard and De Lisi [23].

Similar findings should prompt the researcher to investigate accurately sexual life and hypersexuality of such individuals, in order to design a better treatment plan for these PD patients and also in order to prevent future violent sexual acts.

## Conclusions

The presence of either a pathological personality trait or a PD increases the likelihood of experiencing sexual and behavioral symptoms. This in turn may be attributed to a common element, i.e., the trauma. Traumatic life experiences, especially during childhood, nurture personality development together with the sexual functioning. This should be taken into high consideration both in research and in the clinical setting.

## Supplementary Information

Below is the link to the electronic supplementary material.

Supplementary file1 (DOCX 17 KB)

## Data Availability

The data used for the present manuscript were compiled by the authors based on the studies identified in the systematic review. For our systematic review, we searched MEDLINE and PsycInfo databases.
