# Supplementary material for: Sexual Dysfunctions and Problematic Sexuality in Personality Disorders and Pathological Personality Traits: A Systematic Review
Source: Curr Psychiatry Rep. 2023 Feb 4;25(3):93–103. doi: 10.1007/s11920-023-01409-9 (PMC9968244; doi:10.1007/s11920-023-01409-9)
Supplement: Supplementary file 1 — Supplementary file1 (DOCX 17 KB) [file 11920_2023_1409_MOESM1_ESM.docx]

Ciocca et al. submitted a systematic review about sexual dysfunctions and problematic sexuality in personality disorders and maladaptive personality traits. Sexual dysfunctions are a clinically highly relevant, but oftentimes neglected condition amongst patients with personality disorders (PD). Therefore, a systmatic review about the most recent research is of great importance and impact to the field of clinical psychology and psychiatry and will possibly be read by a large readership.
I very much appreciate the systematic character of this manuscript. The authors followed the PRISMA guidelines and provide clear in- and exclusion criteria according to which they reviewed the most recent literature and summarized its results.
Having said this, I would recommend a thorough major revision in order to improve the readability, but also the impact of the paper.
Specific comments and recommendations:

**1.** Please rewrite the abstract. It is not very meaningful, and the reader does not gain any information except that sexual dysfunctions are frequent and might be related to sexual trauma in PD.

**We thank the reviewer for the comment, we edited part of abstract.**

**2.** Introduction: please rewrite the first parts of the introduction. The definition of PD is rather vage and not state-of-the-art. It might make sense to relate to the DSM-5 AMPD and ICD-11. Also, maybe provide definitions of sexual dysfunctions and sexual risk behaviors.

**We thank the reviewer for the kind observation. We provided the mentioned definitions in the introduction section of the manuscript.**

**3.** Methods: please provide more details on what is meant with "those studies were defined as not relevant to the study topic".

**Thank you very much. We included the suggested element to the “Exclusion Criteria” section of the manuscript.**

**4.** Inclusion of sexual abuse in PD studies: I am not sure how this fits to the general topic of this paper. I would only include studies on the consequences of sexual abuse and early trauma on PD if these also include correlations with sexual dysfunctions or sexual risk behavior. Otherwise I would leave them out. It is well known that early trauma is an important risk factor for PD (and psychopathology in general), thus, the purpose of including these studies if they did not investigate an effect on sexual dysfunctions in PD remains unclear to me.

**We thank the reviewer for this consideration. We agree with your suggestion. Nevertheless, we find sexual trauma as a relevant part of our review article. We believe sexual trauma plays a structuring role for the personality and sexual behavior. We have described this in experimental articles concerning problematic sexuality (see Ciocca G. et al, 2020; Ciocca G, et al 2021). Furthermore, it was observed that sexual abuse and sexual trauma impact both directly and indirectly on sexual behavior. Lastly, being this a systematic review we had to strictly adhere to studies found on our search strategy. In other word, also the sexual dysfunctions, *strictu sensu*, are indirectly related to traumatic sexual experiences. Therefore, we decided to insert the sexual abuse in our paper as a comorbid and etiopathogenetic factor related to problematic sexuality.**

**5**. It would be great to provide some information about how specific these findings are for PD (compared to general population or other clinical groups) and if other factors could explain parts of the data:

could generally increased high risk behavior explain sexual risk behavior? Could medication or comorbid depression explain reduced interest in sexual behavior? What effect does a comorbid PTSD diagnosis have on sexual behavior? How is sexual behavior related to emotional instability, impulsivity, non-suicidal self-injury? Are there any data on effects of psychotherapy on sexual functions?

**We thank the reviewer for the comments and the questions asked. We very much appreciated them. Firstly, we would like to let the reviewer know that because of the systematic nature of our review, We had to strictly respect the search terms. Some of the matters you rightfully mentioned were not fully discussed indeed.**

**Regarding the role of increased risk behavior, we mentioned in the manuscript that there is a plausible association with the increased levels of sexual risk behavior.**

**About the role of medication and comorbid psychopathology, although several studies have investigated the topic, the evaluation of the role of pharmacological treatment on sexual behavior was beyond the purpose of the present review. However, we consider the matter of importance and to be given as a possible future direction for research.**

**In regard to the role of comorbid PTSD diagnosis, we have highlighted several times in the study the role of trauma or PTSD on sexuality. Particularly, we discussed how trauma has an effect on problematic and compulsive sexual behavior.**

**Because of the systematic nature of our review, we did not find any eligible research expressing more thoroughly the relationship between SB and emotional instability etc. however, this association may be considered as a future direction for research.**

**Concerning the effects of psychotherapy on specific sexual dysfunction we noted that experimental studies are lacking. However, there are studies on combined effects on premature ejaculation, for instance, between drugs and psychological treatment. In fact, we could safely say that the effect of psychotherapy on personality could improve sexual problems. Historically, sex therapy by Masters and Johnson are often applied in sexological field.**

**General remarks:
1.** Sometimes the language is difficult to understand.

**We thank the reviewer for the observation. We edited some sentences to allow the reader to fully and clearly understand the manuscript.**

**2.** Please, please drop as many appreviations as possible!!

**Thank you. We reduced the number of unnecessary abbreviations.**
